# Supplementary material for: The practice of early mother-newborn skin-to-skin contact after delivery of healthy term neonate and associated factors among health care professionals at health facilities of Southwestern Oromia, Ethiopia: A cross-sectional study
Source: PLoS One. 2022 Dec 14;17(12):e0274594. doi: 10.1371/journal.pone.0274594 (PMC9750000; doi:10.1371/journal.pone.0274594)
Supplement: S1 File — (DOCX) [file pone.0274594.s001.docx]

**ENGLISH VERSION QUESTIONNAIRE AND CHECKLIST**

**The practice of early mother-newborn skin-to-skin contact after delivery of healthy term neonate and associated factors among health care providers at health facilities of Southwestern Oromia, Ethiopia 2017**

**Greeting:**

Hello, My name is_____________________. I am here today to collect data on Assessment of Practice of early mother-newborn skin-to-skin contact (SSC) after delivery of healthy term neonate and associated factors among health care providers at health facilities of Southwestern Oromia, Ethiopia 2017. The purpose of this study is to explore and describe about health care providers’ practice related to early SSC and its associated factors. I request you to take part in this study and to respond genuinely.

Your cooperation and willingness is greatly helpful in identifying problems related to early SSC in mothers who gave birth. The study will be conducted through self-administer questionnaire and you are being asked for a little of your time, about 20 min, to help us in this study.

Your name will not be written in this form and will never be used in connection with any information you will tell us. There is no possible risk associated with participating in this study except the time spent for responding to the questionnaire. All information given by you will be kept strictly confidential. Your participation will be voluntary and you are not obligated to answer any question you do not wish to answer. If you feel discomfort with the question, it is your right to drop it any time you want. If you have questions regarding this study or would like to be informed of the results after its completion.

Could I have your permission to continue?

1. If yes, will continue to distribute the questionnaire.

2. If no, skip to the next participant by writing reasons for his/her refusal

Informed consent Certified by

Data collectors Name--------------------------------signature-------------------

Date of Data collection-----------------Time started---------------------- Time completed----------

Result of data collection:

1. Completed---------

2. Respondent not available--------

3. Refused------

4. Partially completed.........

Checked by..............................................................

**ANNEX-A: CONSENT FORM AND QUESTIONNAIRE**

**Consent form before distributing the questionnaire**

I have read the information sheet and understood it. I understand what participation in the study means for me. I understand that the information regarding me that is collected in the course of this study will remain confidential. I understand that I am free to take part in the study or refuse, and that I can withdraw from the study at any time. Deciding not to take part or to withdraw from the study will not affect the care that I am normally entitled to. I have had a chance to ask questions and have them answered. This form has been read by _ _ _ _ _ _ _ _ _ _ _ _ _ (Write name of volunteer) and understood the detail of what is written and he/she has freely agreed to take part in the study.

Signature of field worker: ----------------------Name of field worker: --------------------------Date: /…………/……………………

**QUESTIONNAIRE**

**I. Socio –Demographic Data**

1.1. Age (in years)_________

1.2. Sex: a. Male b. Female

1.3. Marital Status: a. Never Married b. Married c. Divorced d. Widowed e. Legally Separated

1.4. Educational level (mark the highest degree held): a. Diploma b. BSc degree c. MSc degree

1.5. Years of practice in delivering babies__________

1.6. Religion a. Orthodox b. Protestant c. Muslim d. Catholic e. Others

1.7. Ethnicity a. Oromo b. Amhara c. Tigre d. Others

1.8. Have you taken any training on newborn care or on skin to skin contact? a. yes b. no

**II. Knowledge about mother-newborn skin to skin contact**

2.1. Which one is the correct procedure to put mother-newborn skin to skin contact?

a. Wrap the newborn with warm cloth and put at the side of the mother or on the mother’s abdomen.

b. Put naked newborn belly-down on his or her mother’s or father’s bare abdomen or chest, allowing contact with nipple, then wrap both with warm cloth and cap for newborn.

2.2. What is the appropriate time to start mother-newborn skin to skin contact?

a. Within the first 1hour b. Within the first 6hours

2.3. What is the minimum time duration for the newborn to stay on skin to skin contact continuously? A.30minutes b. 1hour

2.4. Is skin to skin contact possible during managing complications aroused from labor and delivery like repairing of episiotomy and cervical or perineum tears? A. Yes b. No

2.5. Is skin to skin contact prevents neonatal hypothermia? A. Yes b. No

2.6. Is skin to skin contact promotes effective BF? A. Yes b. No

2.7. Skin to skin contact improves neonatal breathing, prevents neonatal infection, accelerates involution and prevents PPH? a. Yes b. No

**III. Question for those who were not practiced mother-newborn SSC for healthy mothers and uncomplicated delivery.**

3.1. What is your reason for not practicing mother-newborn skin-to-skin contact during the first one hour of birth?

1. Keeping under the heater is a routine practice
2. After wrapping the newborn putting on the mother's abdomen is the right procedure
3. Keeping under the heater is more advantageous than putting skin-to-skin contact
4. Skin-to-skin contact is used only for breastfeeding; so, no problem if breastfeeding delay even for more than an hour
5. Other__________________________________________

**ANNEX-B: Checklist**

| **S.no** | **Questions** |  | |
| --- | --- | --- | --- |
|  | 1. **MATERNAL AND OBSTETRIC FACTORS** | **Yes** | **No** |
| 1.1 | Was there any complication during delivery (like episiotomy, cervical or  Perineum tears)? |  |  |
| 1.2 | Was maternal condition healthy (free from severe illnesses like hypertension, DM)? |  |  |
| 1.3 | Was the mother refused practice of skin-to-skin contact? |  |  |
| 1.4 | Was the mother request to have her baby with her within the first 1hr after birth? |  |  |
| 1.5 | Was the mother positively responded for her request to have her baby with her? |  |  |
|  | 1. **PRACTICE** |  |  |
| 2.1 | Was naked newborn put belly-down on his or her mother’s bare abdomen or chest  and then wrap both with warm cloth, with in the 1^st^hr? |  |  |
| 2. 2 | Was the newborn stays ≥1hr in skin to skin contact continuously? |  |  |
| 2.3 | If Was the newborn allowed to contact with the nipple? |  |  |
| 2.4 | Was the newborn’s head covered with warm cloth or with cap? |  |  |
| 2.5 | Was the newborn wrapped with warm cloth and put on the mother’s abdomen  within the 1^st^ hr? |  |  |
| 2.6 | Was the newborn wrapped with warm cloth and put at the side of the mother on  the bed? |  |  |
| 2.7 | Was the newborn wrapped with warm cloth and/or stay more than an hour under the heater? |  |  |
