# Supplementary material for: The practice of early mother-newborn skin-to-skin contact after delivery of healthy term neonate and associated factors among health care professionals at health facilities of Southwestern Oromia, Ethiopia: A cross-sectional study
Source: PLoS One. 2022 Dec 14;17(12):e0274594. doi: 10.1371/journal.pone.0274594 (PMC9750000; doi:10.1371/journal.pone.0274594)
Supplement: S2 File — (DOCX) [file pone.0274594.s002.docx]

**LOCAL LANGUAGE VERSION /AFAAN OROMO**

**Af-Gaaffii Ogeessota Fayyaa Dhaabbata Fayyaa Hojjataniif Tajaajila Waa’ee Daa’imman Amma Reefu Dhalataniif Tajaaila Ho’a Qaama qaama haadhaa waliin wal tuqsiisuun kennamu irratti Ogeessa Fayyaatiin Kennamuu Fi Wantoota Isaan Walqabatan Irratti Bara 2017 Kibba Lixa Oromiyaatti Qophaa’e.**

**Greeting:**

Akkam jirtu, Maqaan koo_____________________. Har’a kanin asitti argameef odeeffannoo waa’ee daa’imman amma reefu dhalataniif tajaajila ho’a qaamaa ogeessa fayyaatiin kennamuu fi wantoota isaan walqabatan irratti bara 2017 Kibba lixa oromiyaatti dhaabbata fayyaatti kennamu ilaalchisee odeeffannoo funaanuuf. Kayyoon qorannoo kanaas waayee tajaajila ogeessa fayyaatiin daa’imman amma reefu dhalataniif kennamuu fi wantoota isaan walqabatan irratti hubannoo kennuu fi ibsuudha. Ani kanin si gaafachuu barbaades hubannoo gaariidhaan qorannoo kana irratti akka hirmaattudha. Ati na gargaruun kee fi fedhi-qabeessa/ttii tahuun kee rakkoolee tajaajila ogeessa fayyaatiin daa’imman amma reefu dhalataniif kennamu adda baafachuuf gargaara.

Qorannoon kun waraqaan af-gaaffii siif kennameeti yeroo xiqqoo fudhaachun guutta, yoo baay’ate daqiiqaa 20 sitti fudhachuu danda’a.

Asirratti maqaan kee hin ibsamu akkasumas odeeffannoon ati nuuf kennitu maqaa keetiin wal hin qabatu. Yeroo hirmaannaaf sitti fudhatuun alatti, qorannoo kana irratti hirmaachuu keetiin rakkoon tokkolle sirra hin gahu. Odeeffannoon ati naaf kennite hundi icciitiin isaa akka siif eegamu waadaan siif gala.

Hirmaannan kee fedhii irratti kan hudaa’ee fi gaaffii gaafatamte hunda deebiisuuf hin diraqamsiifamtu. Yoo sitti toluu baate gaafannoo kana adda kutuuf mirga guutuu qabda. Waa’ee qorannoo kanaa ilaachisee gaaffii kamiyyuu yoo qabaatte gaafachuu dandeessa.

Gaafatamuuf eeyyamamadha ……………. A. Eeyyee B. Lakkii

Maqaa Hospitaala ……………………

Lakk Koodii ………………………….

Bu’a Gaafficha

1. Xumurameera

2. Hinargamneguyyagaafano

3. Nidide

4. Gar tokkon

Kan Mirkaneesse……………………

**Unkaa waliigaltee**

Odeeffannoo waraqaa kana irra jiru dubbisee hubadheera. Kanin hubadhes hirmaannaan ani qorannoo kana irratti qabu maal akka ta’edha. Odeeffannoon narraa fudhatamus icciitiin isaa akka eegamu hubadheera. Akkasumas ani qorannoo kana irratti yeroon hirmaadhu fedhii fi bilisaan, yeroo barbaachisaa ta’ettis adda kutuu akkan danda’u hubadheera. Hirmaannaa diduun koo yookaan adda kutuun koo tajaajilan ani maamiltoota koof kennu irratti dhiibbaa tokkollee hin qabu. Gaaffiiwwan gaaffachuus tahee deebisuus akkan dandahu hubadheera.

Guchni kun kan dubbifame______________________ (Maqaa nama fedhiin hirmaate barreessi).

Maqaa fi Mallattoo nama odeeffannoo kana funaanee: ----------------------: ---------

Guyyaa:… / ……/……

**I. Kutaa1ffaa Gaaffiwwan Jireenya Hawasumma Ilaalatan (itti mari ykn bakka kenname irrati barreessi)**

1.1. Age (in years)_________

1.2. Saala: a. Dhiira b. Dhalaa

1.3. Haala fuudhaaf heerumaa: a. Hin heerumne b. Heerumeera c. Nan hiike d. Na jalaa du’e e. Seeraan adda baane

1.4. Sadarkaa barumsa: a. Diploma b. BSc degree c. MSc degree

1.5. Muuxannoo deessisuudhaan qabdu waggaa__________

1.6. Amantii: a. Orthodox b. Protestant c. Muslim d. Catholic e. Kan biraa

1.7. Sabummaa a. Oromoo b. Amhaara c. Tigree d. Kan biraa

1.8. Leenjii waa’ee daa’imman amma reefu dhalataniif tajaaila ho’aa qaamaa kennuu irratti fudhatteettaa? a. Eeyyee b. Lakki

**II. Kutaa2ffaa Gaaffiwwan waa’ee beekumsa ogeessa fayyaa Ilaallatan (itti mari ykn bakka kenname irratti barreessi)**

2.1. waa’ee ho’a qaamaa daa’ima reefu dhalatuuf kennuuf kallattiin gogaa haadhaan wal tuqsiisuun isa kamtu sirriidha?

a. Daa’ima uffata ho’aan qoorsanii haadha bukkee kaa’uu yookaan garaa haadhaa irra kaa’uu.

b. Daa’ima qullaa isaa / ishii garaa haadhaa / abbaa irra kaa’uu, fiixee harma haadhaatiin akka wal tuqu gochuun fi uffata ho’aa fi aguugduu mataatti fayyadamanii aguuguu.

2.2. Yeroo inni sirriin daa’imaaf ho’i haadhaan wal tuqsiisuun kennamu kami?

a. Sa’aatii 1 keessatti b. Sa’aatii 6 keessatti

2.3. Daa’imaaf ho’i haadhaan wal tuqsiisuun kennamu kami walitti fufiinsaan hammamiif ta’a?

A. Daqiiqaa 30 b. Sa’aatii 1

2.4. Rakkoolee wal xaxaan yeroo dahuumsaa osoo jiruu fkf qaama dahuumsaa fi mormi gadameessaa cite osoo suphanii daa’imaan amma dhalataniif ho’i haadhaan wal tuqsiisuun kennamu walitti fufiinsaan kennamuu ni danda’amaa? A. Eeyyee b. Lakki

2.5. Ho’i haadhaan wal tuqsiisuun daa’imaan amma dhalataniif kennamu hadooduu qaamaa dhorka? A. Eeyyee b. Lakki

2.6. Ho’i haadhaan wal tuqsiisuun daa’imaan amma dhalataniif kennamu dfanii harma akka hodhaniif haala mijeessa? A. Eeyyee b. Lakki

2.7. Ho’i haadhaan wal tuqsiisuun daa’imaan amma dhalataniif kennamu afuura baafannaa ni fooyyessaa, infectionii daa’imaan amma dhalatanii ittisa, Gadaameessi dafee akka bakkatti deebi’a taasissa akkasumas dhiiga garmalee dahumsa booda bahu dhorka? A. Eeyyee b. Lakki

**III. Kutaa 3ffaa Gaaffiwwan waa’ee Sababa ogeessi fayyaa ho’a haadhaan wal tuqsiisuun daa’imaan amma dhalataniif kennamu hin kennineef Ilaallatan (itti mari ykn bakka kenname irratti barreessi)**

3.1. Sa’aatii tokko keessatti maaliif ho’a haadhaan wal tuqsiisuun daa’imaan amma dhalataniif kennamu hin kennine?

1. Yeroo mara meeshaa ho’a kennu jala kaa’uun sirriidha.
2. Mucaa uffataan maraanii garaa haadhaa iirra kaa’uun sirriidha.
3. Meeshaa ho’a kennu jala kaa’uun ho’a haadhaan wal tuqsiisuun daa’imaan amma dhalataniif kennamu irra sirriidha.
4. Ho’i haadhaan wal tuqsiisuun daa’imaan amma dhalataniif kennamu dafanii harma akka jalqabaniif qofa gargaara.
5. Kan biraa__________________________________________

**ANNEX-B: GUCA (Checklist)**

| **S.no** | **Gaaffiilee** |  | |
| --- | --- | --- | --- |
|  | 1. **MATERNAL AND OBSTETRIC FACTORS** | **Eeyyee** | **Lakki** |
| 1.1 | Rakkoolee wal xaxaan yeroo dahumsaa mudatan jiruu? fknf: tarsa’uu  qaama da’uumsaa, morma gadameessaa |  |  |
| 1.2 | Haalli haadhaa fayya qabeessaa (dhukkuboot akka dhiibbaa dhiigaa fi sukkaara  irraa bilisaa)? |  |  |
| 1.3 | Haati Ho’a haadhaan wal tuqsiisuun daa’imaan amma dhalataniif kennamu  Ni didde? |  |  |
| 1.4 | Daa’imni ishee akka sa’aatii jalqabaa ishee waliin turuuf gaafatteettii? |  |  |
| 1.5 | Haati Ho’a haadhaan wal tuqsiisuun daa’imman amma dhalataniif kennamu gaafatamtee miira gaariin tole jettee? |  |  |
|  | 1. **PRACTICE** |  |  |
| 2.1 | Daa’imni reefu dhalatu ufftaan ho’aan erga qoorfamee booda dhaltee sa’aatii 1 keessattti kallattiin gogaa haadhaan wal tuqsiisuun garaa haadhaa irra kaa’ameera? |  |  |
| 2. 2 | Daa’imni reefu dhalatu kallattiin gogaa haadhaan wal tuqsiifamee itti fufiinsaan  garaa haadhaa irra sa’aatii 1 tureera? |  |  |
| 2.3 | If Daa’imni reefu dhalatu harma haadhaa akka qabatu taasifameera? |  |  |
| 2.4 | Mataan daa’ima reefu dhalatu uffata ho’aan ykn aguugduu matatiin aguugameeraa? |  |  |
| 2.5 | Daa’imni reefu dhalatu uffata ho’aan qoorsamee kallattiin gogaa haadhaa irra  kaa’ameeraa? |  |  |
| 2.6 | Daa’imni reefu dhalatu uffata ho’aan qoorsamee kallattiin haadha bukkee sireerra  kaa’ameeraa? |  |  |
| 2.7 | Daa’imni reefu dhalatu uffata ho’aan qoorsamee sa’aatii tokkoof meeshaa ho’a kennu  jala kaa’ameeraa? |  |  |
