# Supplementary material for: The practice of early mother-newborn skin-to-skin contact after delivery of healthy term neonate and associated factors among health care professionals at health facilities of Southwestern Oromia, Ethiopia: A cross-sectional study
Source: PLoS One. 2022 Dec 14;17(12):e0274594. doi: 10.1371/journal.pone.0274594 (PMC9750000; doi:10.1371/journal.pone.0274594)
Supplement: Attachment — Submitted filename: Response to Reviewers PDF.pdf [file pone.0274594.s003.pdf]

Revision for Reviewers & Academic Editor

1. Please ensure that your manuscript meets PLOS ONE's style requirements, including those for file naming. (Ensured)
2. Please amend your current ethics statement to address the following concerns: Please explain why written consent was not obtained, how you recorded/documented participant consent:

   Answer: it is optional to use the written or verbal consent.

   For this study verbal consent was used because the participants permit to take only verbal consent and the consent taken by reading the format prepared with the questioner and checklist, i.e. ‘’ the data collectors has been read the informed consent for the participants by informing them about benefit, procedures, duration, alternatives of participation (whether participated or not) and confidentiality. By asking the participants the question ‘’could I have your permission to continue?’’

   1. If yes, will continue to distribute the questionnaire.

   2. If no, skip to the next participant by writing reasons for his/her refusal.

3. Please include additional information regarding the checklist and questionnaire used in the study and ensure that you have provided sufficient details that others could replicate the analyses. For instance, if you developed a questionnaire as part of this study and it is not under a copyright more restrictive than CC-BY, please include a copy, in both the original language and English.

Answer: Self-administered questionnaire and observational checklist were developed by reviewing different related literatures, and prepared in English.

<u>The following is the copy of questionnaire and Checklist</u>

Annexes:

<u>ENGLISH VERSION QUESTIONNAIRE AND CHECKLIST</u>

Practice of early mother-newborn skin-to-skin contact after delivery of healthy term neonate and associated factors among health care providers at health facilities of Southwestern Oromia, Ethiopia 2017.

Greeting:

Hello, My name is_______________________. I am here today to collect data on Assessment of Practice of early mother-newborn skin-to-skin contact (SSC) after delivery of healthy term neonate

and associated factors among health care providers at health facilities of Southwestern Oromia, Ethiopia 2017. The purpose of this study is to explore and describe about health care providers' practice related to early SSC and its associated factors. I request you to take part in this study and to respond genuinely.

Your cooperation and willingness is greatly helpful in identifying problems related to early SSC in mothers who gave birth. The study will be conducted through self-administer questionnaire and you are being asked for a little of your time, about 20 min, to help us in this study.

Your name will not be written in this form and will never be used in connection with any information you will tell us. There is no possible risk associated with participating in this study except the time spent for responding to the questionnaire. All information given by you will be kept strictly confidential. Your participation will be voluntary and you are not obligated to answer any question you do not wish to answer. If you feel discomfort with the question, it is your right to drop it any time you want. If you have questions regarding this study or would like to be informed of the results after its completion.

Could I have your permission to continue?

1. If yes, will continue to distribute the questionnaire.
2. If no, skip to the next participant by writing reasons for his/her refusal

Informed consent Certified by

Data collectors Name-------------------------------signature------------------

Date of Data collection-----------------Time started--------------------- Time completed----------

Result of data collection:

1. Completed---------
2. Respondent not available--------
3. Refused------
4. Partially completed.........

Checked by...

ANNEX-A: CONSENT FORM AND QUESTIONNAIRE

Consent form before distributing the questionnaire

I have read the information sheet and understood it. I understand what participation in the study means for me. I understand that the information regarding me that is collected in the course of this study will remain confidential. I understand that I am free to take part in the study or refuse, and that I can withdraw from the study at any time. Deciding not to take part or to withdraw from the study will not affect the care that I am normally entitled to. I have had a chance to ask questions

and have them answered. This form has been read by _ _ _ _ _ _ _ _ _ _ _ _ _ _ (Write name of volunteer) and understood the detail of what is written and he/she has freely agreed to take part in the study.

Signature of field worker: ---------------------Name of field worker: -------------------------Date: /………../……………………

QUESTIONNAIRE

I. Socio –Demographic Data

1.1. Age (in years)_________

1.2. Sex: a. Male b. Female

1.3. Marital Status: a. Never Married b. Married c. Divorced d. Widowed e. Legally Separated

1.4. Educational level (mark the highest degree held): a. Diploma b. BSc degree c. MSc degree

1.5. Years of practice in delivering babies__________

1.6. Religion a. Orthodox b. Protestant c. Muslim d. Catholic e. Others

1.7. Ethnicity a. Oromo b. Amhara c. Tigre d. Others

1.8. Have you taken any training on newborn care or on skin to skin contact? a. yes b. no

II. Knowledge about mother-newborn skin to skin contact

2.1. Which one is the correct procedure to put mother-newborn skin to skin contact?

a. Wrap the newborn with warm cloth and put at the side of the mother or on the mother's abdomen.

b. Put naked newborn belly-down on his or her mother's or father's bare abdomen or chest, allowing contact with nipple, then wrap both with warm cloth and cap for newborn.

2.2. What is the appropriate time to start mother-newborn skin to skin contact?

a. Within the first 1hour b. Within the first 6hours

2.3. What is the minimum time duration for the newborn to stay on skin to skin contact continuously? A.30minutes b. 1hour

2.4. Is skin to skin contact possible during managing complications aroused from labor and delivery like repairing of episiotomy and cervical or perineum tears? A. Yes b. No

2.5. Is skin to skin contact prevents neonatal hypothermia? A. Yes b. No

2.6. Is skin to skin contact promotes effective BF? A. Yes b. No

2.7. Skin to skin contact improves neonatal breathing, prevents neonatal infection, accelerates involution and prevents PPH? a. Yes b. No

III. Question for those who were not practiced mother-newborn SSC for healthy mothers and uncomplicated delivery.

3.1. What is your reason for not practicing mother-newborn skin-to-skin contact during the first one hour of birth?

a. Keeping under the heater is a routine practice
b. After wrapping the newborn putting on the mother's abdomen is the right procedure
c. Keeping under the heater is more advantageous than putting skin-to-skin contact
d. Skin-to-skin contact is used only for breastfeeding; so, no problem if breastfeeding delay even for more than an hour
e. Other____

ANNEX-B: Checklist

S.no	Questions		
	**1. MATERNAL AND OBSTETRIC FACTORS**	**Yes**	**No**
1.1	Was there any complication during delivery (like episiotomy, cervical or Perineum tears)?		
1.2	Was maternal condition healthy (free from severe illnesses like hypertension, DM)?		
1.3	Was the mother refused practice of skin-to-skin contact?		
1.4	Was the mother request to have her baby with her within the first 1hr after birth?		
1.5	Was the mother positively responded for her request to have her baby with her?		
	**2. PRACTICE**		
2.1	Was naked newborn put belly-down on his or her mother’s bare abdomen or chest and then wrap both with warm cloth, with in the 1sthr?		
2. 2	Was the newborn stays ≥1hr in skin to skin contact continuously?		
2.3	Was the newborn allowed to contact with the nipple?		
2.4	Was the newborn’s head covered with warm cloth or with cap?		
2.5	Was the newborn wrapped with warm cloth and put on the mother’s abdomen within the 1st hr?		
2.6	Was the newborn wrapped with warm cloth and put at the side of the mother on the bed?		
2.7	Was the newborn wrapped with warm cloth and/or stay more than an hour under the heater?		

4. Please state whether you validated the questionnaire prior to testing on study participants. Please provide details regarding the validation group within the methods section.

Answer: This questionnaire was tested for validity by the advisors and other professional's feedbacks were integrated into the final tool used for the data collection.

5.Thank you for stating the following in the Acknowledgments/Funding Section of your manuscript: The entire necessary costs (material and humanitarian) for the study were covered by Ethio-Canada MCH project funded this study.‖

We note that you have provided funding information that is not currently declared in your Funding Statement. However, funding information should not appear in the Acknowledgments section or other areas of your manuscript. We will only publish funding information present in the Funding Statement section of the online submission form.

Please remove any funding-related text from the manuscript and let us know how you would like to update your Funding Statement. Currently, your Funding Statement reads as follows:

―The author(s) received no specific funding for this work.‖

Please include your amended statements within your cover letter; we will change the online submission form on your behalf. ***Corrected to***: *the author(s) received no specific funding for this work*

6. Please note that in order to use the direct billing option the corresponding author must be affiliated with the chosen institute. Please either amend your manuscript to change the affiliation or corresponding author, or email us at plosone@plos.org with a request to remove this option.

7. ORCID: 0000000322665006.

8. Please include your full ethics statement in the Methods 'section of your manuscript file. In your statement, please include the full name of the IRB or ethics committee who approved or waived your study, as well as whether or not you obtained informed written or verbal consent. If consent was waived for your study, please include this information in your statement as well. Included.

RESPONSES FOR REVIEWER'S COMMENTS

1. **Reviewer #1:** • How did you measure the practice? Relevant results should be stated

Answer: If the providers apply early skin-to-skin contact according to the checklist used for the study which contains 7 questions. (*1. Was naked newborn put belly-down on his or her mother's bare abdomen or chest and then wrap both with warm cloth, with in the 1st hr? 2. Was the newborn stays ≥1hr in skin to skin contact continuously? 3. Was the newborn allowed to contact with the nipple? 4. Was the newborn's head covered with warm cloth or with cap? 5. Was the newborn wrapped with warm cloth and put on the mother's abdomen within the 1st hour? 6. Was the newborn wrapped with warm cloth and put at the side of the mother on the bed? 7. Was the newborn wrapped with warm cloth and/or stay more than an hour under the heater*?).

• Explain more about tools and Validity and reliability of the tools: This questionnaire was tested for validity by the advisors and other professional's feedbacks were integrated into the final tool used for the data collection.

• Remove results (For example (P=0.002, AOR=4, CI=1.7, 10…) from the discussion section: Removed

• You can use the following articles in the introduction and discussion sections: *some used in introduction and some in discussion.*

Reviewer #2: General: This is an interesting study that focuses on the practice of skin to skin contact post-delivery among new mothers in Ethiopia.

Introduction: Although the authors mentioned about Ethiopia's maternal care. However, the authors should elaborate on the ***national policy and maternal care services.*** The world prevalence is not very important in this case; rather the *reader would be more interested on Ethiopia's national policy on maternal delivery and care;* as well as the maternity services / labor services available. Why is 90% of birth occurring at home? is that cultural practice or due to insufficient maternity hospital? Transport or distance to hospital? Suggest to focus solely on Ethiopia, describing its maternal healthcare services, national policy, home birth rate and why it is so prevalent and then on the practices of skin-to-skin contact post-delivery in Ethiopia; Also, to add if other similar studies have been done in Ethiopia and what are the current known findings (if any): as a result of insufficient health care facilities.

Statement of the problem: paragraph 5 - this paragraph is not clear in terms of what the authors are saying. Please reword the sentences: corrected

Method: please explain why convenience sampling was applied? The individual participants were few in number and was not suitable to use probable sampling techniques. That was the reason why we preferred to use all participants those illegible and willing to participate in our study.
Also, the method states that pre-testing was done. What kind of pre-testing? *Direct questioning and observational pretest implemented.* Was reliability of the questionnaire measured? What was the result of the pre-testing? This questionnaire was tested for validity by the advisors and other professional's feedbacks were integrated into the final tool used for the data collection. And amendments had done.

In data collection, it stated that the healthcare providers were unaware they were being observed however, consent was taken before they were recruited so, how was that achieved? Please explain. The observation done without the knowledge of participants or they did not had an idea about the exact care being observed after taking the consent for assessment of general immediate new born care. If they had information about what the data collectors observed they will try to apply SSC what they were not did before.
Results: Please check table 3 maternal illnesses if your significance level is correct? Checked & corrected
Discussion: could be better written at the end to tally all the four factors and how it can be used to improve the skin to skin practice in Ethiopia: Corrected
