# Supplementary material for: The practice of early mother-newborn skin-to-skin contact after delivery of healthy term neonate and associated factors among health care professionals at health facilities of Southwestern Oromia, Ethiopia: A cross-sectional study
Source: PLoS One. 2022 Dec 14;17(12):e0274594. doi: 10.1371/journal.pone.0274594 (PMC9750000; doi:10.1371/journal.pone.0274594)
Supplement: Attachment — Submitted filename: Response to Reviewers.docx [file pone.0274594.s004.docx]

**Tittle**: ***The Practice of early mother-newborn skin-to-skin contact after delivery of healthy term neonate and associated factors among health care providers at health facilities of Southwestern Ethiopia: A cross-sectional study***

**The responses of reviewed manuscript for academic editor & reviewers**

Academic Editor
**Comment 1:** Please review your reference list to ensure that it is complete and correct. If you have cited papers that have been retracted, please include the rationale for doing so in the manuscript text, or remove these references and replace them with relevant current references. Any changes to the reference list should be mentioned in the rebuttal letter that accompanies your revised manuscript. If you need to cite a retracted article, indicate the article’s retracted status in the References list and also include a citation and full reference for the retraction notice.

**Response**: We have used five references that recommended by the ***reviewer #1*** ***(****Ref: Number 2, 6, 7, 8, 12)*: these are listed below under the response for **reviewer #1.**

**Reviewer#1**:

**Comment 1:** The title and objective is not consistent with the results of the article
Items such as maternal factors are mentioned in the results that are not mentioned in the title or objective.

Response: thank you for your constructive comments.

- Un-necessary factors mentioned in the result like ‘maternal factors’ removed from the result part, which are not related with title and objectives.
  **Comment 2:** The number of references is low, Suggested articles for use in this manuscript.
- I considered the references that you suggested and 5 of them cited ***(****Ref: Number 2, 6, 7, 8, 12*). These are:
- Karimi FZ, Sadeghi R, Maleki-Saghooni N, Khadivzadeh T. The effect of mother-infant skin to skin contact on success and duration of first breastfeeding: A systematic review and meta-analysis. Taiwanese Journal of Obstetrics and Gynecology. 2019 Jan 1; 58(1):1-9.
- Karimi A, Bagheri S, Khadivzadeh T, Mirzaii Najmabadi Kh. The Effect of an Interventional Program, Based on the Theory of Ethology, on Breastfeeding Competence of Infants. Iranian Journal of Neonatology 2014; 5(3): 10-12.
- Karimi FZ, Khadivzadeh T, Saeidi M, Bagheri S. The Effect of Kangaroo Mother Care Immediately after Delivery on Mother-infant Attachment and on Maternal Anxiety about the Baby 3- Months after Delivery: a Randomized Controlled Trial. Int J Pediatr 2016; 4(9): 3561-70.
- Karimi FZ, MIRI HH, Salehian M, Khadivzadeh T, Bakhshi M. The Effect of Mother-Infant Skin to Skin Contact after Birth on Third Stage of Labor: A Systematic Review and Meta-Analysis. Iranian journal of public health. 2019 Apr;48(4):612.
- Khadivzadeh T, Karimi FZ, Tara F, Bagheri S. The Effect of Postpartum Mother–Infant Skin-to-Skin Contact on Exclusive Breastfeeding In neonatal period: A Randomized Controlled Trial. Int J Pediatr 2016; 4(5): 5409-17. DOI: 10.22038/ijp.2016.7522.

**Reviewer#2**:

**Comment 1:** There are several grammatical error of the manuscript, especially in the abstract section that may benefit from copyediting.

Response: Thank you for your positive and constructive comments. As much as possible I tried to correct the whole document for grammatical errors including the abstract part.
