# Supplementary material for: The practice of early mother-newborn skin-to-skin contact after delivery of healthy term neonate and associated factors among health care professionals at health facilities of Southwestern Oromia, Ethiopia: A cross-sectional study
Source: PLoS One. 2022 Dec 14;17(12):e0274594. doi: 10.1371/journal.pone.0274594 (PMC9750000; doi:10.1371/journal.pone.0274594)
Supplement: Attachment — Submitted filename: Response to Reviewers.docx [file pone.0274594.s005.docx]

**Tittle**: ***The Practice of early mother-newborn skin-to-skin contact after delivery of healthy term neonate and associated factors among health care providers at health facilities of Southwestern Ethiopia: A cross-sectional study***

**The responses of reviewed manuscript for academic editor & reviewers**

Academic Editor
**Comment 1:** We noted multiple language issues, especially in Abstract. Please have your manuscript carefully copyedited and correct any language errors this time

**Response**: We have tried to correct the grammatical errors in th abstract section of the manuscript.

**Comment 2:** In addition, we note that one or more reviewers has recommended that you cite specific previously published works. As always, we recommend that you please review and evaluate the requested works to determine whether they are relevant and should be cited. It is not a requirement to cite these works. For instance, we feel that the suggested references 2,5,6,7,8, and 12 are not so relevant and you are optional to cite them.

**Response**: the citations that said irrelevant to our study are removed according to your suggestion.

**Comment 3:** Furthermore, We note that you have indicated that “All relevant data are within the manuscript”. Finally, we noted the following conflicted data statements:
-On the submission details page: "No - some restrictions will apply" and "All relevant data are within the manuscript" -In the manuscript: "The data used or analyzed throughout the current study will be obtained up on request from the corresponding author and coauthors." Please comment on this and keep your data statements consistent.

**Response**: we make data statements consistent according to your comment.

Reviewer #1: ‘’Please use the space provided to explain your answers to the questions above. You may also include additional comments for the author, including concerns about dual publication, research ethics, or publication ethics. (Please upload your review as an attachment if it exceeds 20,000 characters)’’

Reviewer #2: ‘’Dear Author, thank you for preparing the corrections. Improvements have been made to the manuscript and are satisfactory. All comments of the reviewers were answered. Well done!’’

- Thank you the reviewers and editors for your constructive comments and suggestions. We gain so many experiences from your valuable comments and suggestions. Thank you again!
